# Mechanical power during extracorporeal membrane oxygenation and hospital mortality in patients with acute respiratory distress syndrome

**DOI:** 10.1186/s13054-020-03428-x

**Published:** 2021-01-06

**Authors:** Li-Chung Chiu, Shih-Wei Lin, Li-Pang Chuang, Hsin-Hsien Li, Pi-Hua Liu, Feng-Chun Tsai, Chih-Hao Chang, Chen-Yiu Hung, Chung-Shu Lee, Shaw-Woei Leu, Han-Chung Hu, Chung-Chi Huang, Huang-Pin Wu, Kuo-Chin Kao

**Affiliations:** 1 Department of Thoracic Medicine, Chang Gung Memorial Hospital, Chang Gung University College of Medicine, Linkou, No. 5, Fu-Shing St., GuiShan, Taoyuan, Taiwan; 2 Graduate Institute of Clinical Medical Sciences, College of Medicine, Chang Gung University, Taoyuan, Taiwan; 3 Department of Thoracic Medicine, New Taipei Municipal TuCheng Hospital and Chang Gung University, Taoyuan, Taiwan; 4 Department of Respiratory Therapy, Chang Gung University College of Medicine, Taoyuan, Taiwan; 5 Institute of Emergency and Critical Care Medicine, School of Medicine, National Yang-Ming University, Taipei, Taiwan; 6 Clinical Informatics and Medical Statistics Research Center, College of Medicine, Chang Gung University, Taoyuan, Taiwan; 7 Division of Endocrinology and Metabolism, Department of Internal Medicine, Chang Gung Memorial Hospital, Taoyuan, Taiwan; 8 Division of Cardiovascular Surgery, Chang Gung Memorial Hospital, Taoyuan, Taiwan; 9 Department of Respiratory Therapy, Chang Gung Memorial Hospital, Chang Gung University College of Medicine, Taoyuan, Taiwan; 10 Division of Pulmonary, Critical Care and Sleep Medicine, Chang Gung Memorial Hospital, Keelung, Taiwan; 11 Department of Intensive Care, Xiamen Chang Gung Hospital, Xiamen, China

**Keywords:** Mechanical power, Acute respiratory distress syndrome, Extracorporeal membrane oxygenation, Ventilator-induced lung injury, Functional lung size, Compliance, Mortality

## Abstract

**Background:**

Mechanical power (MP) refers to the energy delivered by a ventilator to the respiratory system per unit of time. MP referenced to predicted body weight (PBW) or respiratory system compliance have better predictive value for mortality than MP alone in acute respiratory distress syndrome (ARDS). Our objective was to assess the potential impact of consecutive changes of MP on hospital mortality among ARDS patients receiving extracorporeal membrane oxygenation (ECMO).

**Methods:**

We performed a retrospective analysis of patients with severe ARDS receiving ECMO in a tertiary care referral center in Taiwan between May 2006 and October 2015. Serial changes of MP during ECMO were recorded.

**Results:**

A total of 152 patients with severe ARDS rescued with ECMO were analyzed. Overall hospital mortality was 53.3%. There were no significant differences between survivors and nonsurvivors in terms of baseline values of MP or other ventilator settings. Cox regression models demonstrated that mean MP alone, MP referenced to PBW, and MP referenced to compliance during the first 3 days of ECMO were all independently associated with hospital mortality. Higher MP referenced to compliance (HR 2.289 [95% CI 1.214–4.314], *p* = 0.010) was associated with a higher risk of death than MP itself (HR 1.060 [95% CI 1.018–1.104], *p* = 0.005) or MP referenced to PBW (HR 1.004 [95% CI 1.002–1.007], *p* < 0.001). The 90-day hospital mortality of patients with high MP (> 14.4 J/min) during the first 3 days of ECMO was significantly higher than that of patients with low MP (≦ 14.4 J/min) (70.7% vs. 46.8%, *p* = 0.004), and the 90-day hospital mortality of patients with high MP referenced to compliance (> 0.53 J/min/ml/cm H_2_O) during the first 3 days of ECMO was significantly higher than that of patients with low MP referenced to compliance (≦ 0.53 J/min/ml/cm H_2_O) (63.6% vs. 29.7%, *p* < 0.001).

**Conclusions:**

MP during the first 3 days of ECMO was the only ventilatory variable independently associated with 90-day hospital mortality, and MP referenced to compliance during ECMO was more predictive for mortality than was MP alone.

## Background

Mechanical ventilation remains the cornerstone of management strategies for acute respiratory distress syndrome (ARDS), and extracorporeal membrane oxygenation (ECMO) is widely used as a salvage therapy for refractory hypoxemia in patients with severe ARDS. ECMO allows the lungs to rest and prevents the risk of ventilator-induced lung injury (VILI) by lowering airway pressure, tidal volume (*V*_T_), and FiO_2_. However, the optimal ventilation strategies for patients with severe ARDS receiving ECMO have yet to be defined [1, 2].

Mechanical power (MP) refers to the amount of energy per unit of time transmitted to the respiratory system by a mechanical ventilator, as determined by volume, pressure, flow, and respiratory rate (RR). It is therefore reasonable to assume that MP is superior to single ventilator parameter in predicting the risk of VILI [3, 4]. VILI originates from the interaction between the energy load (i.e.*,* MP) and the pathophysiological characteristics of the lungs (size, homogeneity and recruitability) [4–6]. Therefore, the same MP may have different impact on respiratory system depending on the applied conditions of lungs, and MP should be referenced at least to the functional lung size in order to accurately reflect the actual amount of energy applied to the lungs, i.e.*,* specific power [7–11].

Recent studies have shown that MP is independently associated with in-hospital mortality among critically ill patients [12], and high MP levels have been linked to increased mortality in ARDS patients [13]. However, MP alone does not have better predictive value for patients with ARDS, and it is preferable to adjust MP to predicted body weight (PBW) [7] or respiratory system compliance in terms of well-aerated tissue [8].

ECMO enhanced lung-protective ventilation to mitigate the energy load (i.e*.,* MP) delivered to the respiratory system; however, researchers have yet to contrast the influence of MP alone and MP referenced to functional lung size on the mortality in ARDS patients undergoing ECMO. Our objective in this study was to assess the role of serial changes in MP (adjusted for PBW or compliance) on hospital mortality in patients with severe ARDS undergoing ECMO.

## Methods

### Study design and patients

This study was based on retrospective analysis of patients with severe ARDS who had been treated using ECMO between May 2006 and October 2015 at Chang Gung Memorial Hospital (CGMH) in Taiwan. CGMH is a tertiary care referral center with a 3700-bed general ward and 278-bed adult intensive care unit (ICU) with a high volume of venoarterial and venovenous mode ECMO exceeding 100 cases annually, and only 20% of the indications for ECMO was patients with severe ARDS. Exclusion criteria were as follows: (1) age < 20 years, (2) malignancies with poor prognosis within 5 years, (3) significant underlying comorbidities or severe multiple organ failure refractory to treatment, and (4) mortality within 3 days after ECMO initiation. The local Institutional Review Board for Human Research approved this study (CGMH IRB No. 201600632B0) and waived the need for informed consent.

### Definitions

ARDS was defined in accordance with the Berlin criteria [14]. MP was calculated in accordance with the methods [4] based on *V*_T_, RR, peak inspiratory pressure (Ppeak), and driving pressure (∆*P*) using the following equation:

$$\begin{aligned} {\text{MP}}\left( {\text{Joules/minutes}} \right)\left( {\text{J/min}} \right) &= 0.098 \times {{V}}_{{\text{T}}} \times {\text{RR}}\\&\quad \times ({\text{Ppeak}}{-}{1}/{2} \times\Delta P). \\ {\text{MP}}\,{\text{referenced}}\,{\text{to}}\,{\text{PBW}} &= {\text{MP/PBW}}{.} \\ {\text{MP}}\,{\text{referenced}}\,{\text{to}}\,{\text{compliance}} &= {\text{ MP/Compliance}}{.} \\ \end{aligned}$$

Ppeak is equivalent to plateau pressure in pressure-controlled ventilation [15–18]. Ppeak has been used as a surrogate for plateau pressure to calculate MP if not specified [19], and similar effect of MP on mortality was demonstrated when considering Ppeak instead of plateau pressure for calculating MP [12]. One recent prospective study used dynamic driving pressure (Ppeak minus PEEP) to calculate MP, referring to the measure as dynamic MP [20]. Hospital mortality refers to all-cause death during the hospital stay. Patients who remained alive for 90 days after discharge from the hospital were regarded as survivors.

### Data collection

Demographic data, risk factors for ARDS, underlying comorbidities, Sequential Organ Failure Assessment (SOFA) score, and lung injury score were collected prior to ECMO initiation. The dates of hospital and ICU admission, ARDS onset, mechanical ventilator initiation and liberation, ECMO cannulation and decannulation, ICU and hospital discharge, and time of death were recorded. Arterial blood gas parameters and mechanical ventilator settings were recorded at the time of ECMO initiation and at approximately 10 a.m. on days 1, 2, and 3 after ECMO initiation.

### Statistical analysis

Continuous variables were presented as mean ± standard deviation or median (interquartile range), and categorical variables were reported as numbers (percentages). A student’s *t* test or the Mann–Whitney *U* test was used to compare continuous variables between groups. Categorical variables were tested using the chi-square test for equal proportions or Fisher’s exact test. Paired Student’s t tests were used to compare continuous variables before and after ECMO. Receiver operating characteristic curve and Youden index were used to determine the cutoff to dichotomize continuous variables. Risk factors associated with hospital mortality were analyzed using univariate analysis in the first step, followed by Cox proportional hazard regression model with stepwise selection. The results were presented using the hazard ratio (HR) and 95% confidence interval (CI). Cumulative mortality curves were generated as a function of time using the Kaplan–Meier approach and compared using the log-rank test. All statistical analysis was performed using SPSS 22.0 statistical software, and a two-sided *p* value < 0.05 was considered statistically significant.

## Results

A total of 152 patients with severe ARDS rescued by ECMO were included in the analysis, which examined the impact of MP on hospital mortality. Overall all-cause in-hospital mortality was 53.3%. All patients were deeply sedated and paralyzed, and most cases received pressure-controlled ventilation until attempts at weaning from ECMO. The ECMO techniques didn’t show significant difference during the study period. Hospital mortality was not significantly different between patients in the earlier years and later years of the study period (2006–2011: 77 patients, mortality rate 54.5%; 2012–2015: 75 patients, mortality rate 52%, *p* = 0.753). Patients in the later years received significantly lower *V*_T_, higher PEEP, lower Ppeak, and lower MP during the first 3 days of ECMO than did patients in the earlier years (Additional file 1: Table S1). The mean value of MP from day 1 to day 3 on ECMO didn’t show significant difference (*p* = 0.150), and mean MP during the first 3 days of ECMO was used to evaluate the impact on hospital mortality (Additional file 2: Table S2).

### Comparisons of survivors and nonsurvivors

As shown in Table 1, the mean age of nonsurvivors was higher than that of survivors. Nonsurvivors suffered from ARDS for a longer duration before ECMO initiation, and a higher percentage were immunocompromised. There were no significant differences between the two groups in terms of baseline ventilator settings. After receiving ECMO support, nonsurvivors received significantly higher MP than did survivors, with higher MP referenced to PBW, higher MP referenced to compliance, higher Ppeak, lower dynamic compliance, and higher total RR (all *p* < 0.05). The SOFA scores of nonsurvivors were also significantly higher during the first 3 days of ECMO support.

**Table 1:** Background characteristics and clinical variables: survivors and nonsurvivors

Variables	All	Survivors	Nonsurvivors	*p*
	(*n* = 152)	(*n* = 71)	(*n* = 81)	
Age (years)	50.3 ± 16.4	46.0 ± 16.5	54.1 ± 15.4	0.002
Male (gender)	103 (67.8%)	48 (67.6%)	55 (67.9%)	0.969
Body mass index (kg/m^2^)	25.8 ± 5.3	26.0 ± 5.8	25.6 ± 4.7	0.631
ARDS etiologies				
Pulmonary cause	118 (78%)	59 (83%)	59 (73%)	0.130
Extrapulmonary cause	34 (22%)	12 (17%)	22 (27%)	0.130
Diabetes mellitus	40 (26%)	23 (32%)	17 (21%)	0.111
Chronic liver disease	21 (14%)	6 (9%)	15 (19%)	0.073
Immunocompromised status	40 (26%)	11 (16%)	29 (36%)	0.005
SOFA score before ECMO	10.8 ± 3.2	10.3 ± 3.1	11.3 ± 3.2	0.067
Lung injury score before ECMO	3.4 ± 0.4	3.4 ± 0.4	3.3 ± 0.4	0.106
ARDS duration before ECMO (h)	28 (7–122)	10 (4–64)	54 (17–195)	< 0.001
PaO_2_/FiO_2_ (mm Hg) before ECMO	63 (52–88)	64 (53–80)	63 (52–107)	0.168
Ventilator settings before ECMO				
MP (J/min)	23.8 ± 9.6	24.1 ± 10.3	23.5 ± 9.0	0.668
MP/PBW (× 10^−3^ J/min/kg)	416 ± 172	410 ± 174	423 ± 171	0.645
MP/Compliance (J/min/ml/cm H_2_O)	1.27 ± 0.76	1.21 ± 0.75	1.33 ± 0.78	0.380
Tidal volume (ml/kg PBW)	7.7 ± 2.4	7.7 ± 2.3	7.8 ± 2.5	0.658
PEEP (cm H_2_O)	12.0 ± 2.8	12.2 ± 2.5	11.8 ± 3.0	0.288
Peak inspiratory pressure (cm H_2_O)	33.9 ± 6.5	33.6 ± 6.0	34.2 ± 6.9	0.605
Mean airway pressure (cm H_2_O)	18.6 ± 4.4	18.4 ± 4.2	18.8 ± 4.6	0.588
Dynamic compliance (ml/cm H_2_O)	22.6 ± 11.3	23.7 ± 11.6	21.8 ± 11.1	0.420
Total respiratory rate (breaths/min)	24.0 ± 6.9	23.7 ± 7.4	24.3 ± 6.6	0.596
Spontaneous respiratory rate (breaths/min)	0 (0–7)	1 (0–6)	0 (0–7)	0.982
Minute ventilation (L/min)	10.6 ± 3.8	10.7 ± 4.1	10.5 ± 3.6	0.816
SOFA score from day 1 to day 3 on ECMO	9.6 ± 2.3	8.8 ± 1.9	10.4 ± 2.4	< 0.001
PaO_2_/FiO_2_ (mm Hg) from day 1 to day 3 on ECMO	178 (131–240)	200 (146–247)	165 (124–211)	0.588
Ventilator settings from day 1 to day 3 on ECMO				
MP (J/min)	12.1 ± 6.2	10.9 ± 4.3	13.1 ± 7.4	0.022
MP/PBW (× 10^−3^ J/min/kg)	206 ± 111	185 ± 67	226 ± 137	0.022
MP/Compliance (J/min/ml/cm H_2_O)	0.73 ± 0.46	0.60 ± 0.32	0.86 ± 0.53	< 0.001
Tidal volume (ml/kg PBW)	6.0 ± 2.2	6.1 ± 2.0	6.0 ± 2.4	0.914
PEEP (cm H_2_O)	12.0 ± 3.3	12.3 ± 3.2	11.7 ± 3.3	0.202
Peak inspiratory pressure (cm H_2_O)	31.7 ± 5.6	30.6 ± 5.1	32.8 ± 5.9	0.018
Mean airway pressure (cm H_2_O)	17.7 ± 4.0	17.4 ± 3.6	17.9 ± 4.3	0.406
Dynamic compliance (ml/cm H_2_O)	19.2 ± 8.1	21.1 ± 7.7	17.4 ± 8.1	0.006
Total respiratory rate (breaths/min)	16.0 ± 4.4	15.2 ± 4.1	16.7 ± 4.6	0.035
Spontaneous respiratory rate (breaths/min)	1 (0–4)	0 (0–4)	2 (0–5)	0.114
Minute ventilation (L/min)	5.7 ± 2.8	5.2 ± 2.0	6.0 ± 3.2	0.068

Data are presented as mean ± standard deviation, count or median (interquartile range)

*ARDS* acute respiratory distress syndrome, *ECMO* extracorporeal membrane oxygenation, *FiO*_*2*_ fraction of inspired oxygen, *MP* mechanical power, *PaO*_*2*_ partial pressure of oxygen in arterial blood, *PBW* predicted body weight, *PEEP* positive end-expiratory pressure, *SOFA* Sequential Organ Failure Assessment

### Comparing patients receiving high and low mechanical power

As shown in Table 2, the maximum Youden index value was used to categorize patients according to MP, using a cutoff point of 14.4 J/min during the first 3 days of ECMO: high MP group (41 patients; 27%) and low MP group (111 patients; 73%). No significant differences were observed between the two groups in terms of MP or other ventilator settings variables prior to ECMO initiation. After ECMO support, the high MP and low MP groups differed significantly in all ventilator settings variables except for PEEP and dynamic compliance (all *p* < 0.001). Patients in the high MP group had significantly higher mortality than did patients in the low MP group. As shown in Table 3, the maximum Youden index value was used to categorize patients according to MP referenced to compliance, using a cutoff point of 0.53 J/min/ml/cm H_2_O during the first 3 days of ECMO: high MP/Compliance group (88 patients; 58%) and low MP/Compliance group (64 patients; 42%). Before ECMO initiation, MP/Compliance, not MP alone, was significantly different between the two groups. After ECMO support, the high MP/Compliance and low MP/Compliance groups differed significantly in all ventilator settings variables except for tidal volume. Patients in the high MP/Compliance group had significantly higher mortality than did patients in the low MP/Compliance group.

**Table 2:** Ventilator settings, clinical variables, and outcomes as a function of mechanical power during ECMO

Variables	MP during the first 3 days of ECMO		*p*
	High (*n* = 41) (> 14.4 J/min)	Low (*n* = 111) (≤ 14.4 J/min)	
Ventilator settings before ECMO			
MP (J/min)	25.0 ± 9.5	23.3 ± 9.5	0.339
MP/PBW (× 10^−3^ J/min/kg)	441 ± 166	408 ± 172	0.316
MP/Compliance (J/min/ml/cm H_2_O)	1.32 ± 0.71	1.26 ± 0.78	0.672
Tidal volume (ml/kg PBW)	8.3 ± 2.3	7.5 ± 2.4	0.062
PEEP (cm H_2_O)	11.9 ± 2.7	12.0 ± 2.8	0.786
Peak inspiratory pressure (cm H_2_O)	34.4 ± 6.5	33.8 ± 6.5	0.568
Mean airway pressure (cm H_2_O)	19.2 ± 3.9	18.4 ± 4.5	0.310
Dynamic compliance (ml/cm H_2_O)	22.3 ± 8.4	22.7 ± 12.1	0.869
Total respiratory rate (breaths/min)	23.9 ± 6.7	24.0 ± 7.1	0.891
Spontaneous respiratory rate (breaths/min)	1 (0–6)	0 (0–7)	0.956
Minute ventilation (L/min)	11.2 ± 3.5	10.3 ± 3.9	0.205
Arterial blood gas before ECMO			
pH	7.24 ± 0.16	7.29 ± 0.13	0.056
PaCO_2_ (mm Hg)	56.1 ± 20.0	51.1 ± 18.4	0.150
PaO_2_ (mm Hg)	72.4 ± 33.4	74.5 ± 41.7	0.776
Saturation (%)	83.2 ± 17.4	85.1 ± 14.4	0.508
PaO_2_/FiO_2_ (mm Hg)	66.5 (49.7–85.7)	63 (53–90.7)	0.882
SOFA score before ECMO	11.9 ± 3.1	10.4 ± 3.1	0.013
Ventilator settings from day 1 to day 3 on ECMO			
MP (J/min)	20.3 ± 5.3	9.1 ± 3.0	< 0.001
MP/PBW (× 10^−3^ J/min/kg)	343 ± 117	159 ± 55	< 0.001
MP/Compliance (J/min/ml/cm H_2_O)	1.14 ± 0.48	0.59 ± 0.35	< 0.001
Tidal volume (ml/kg PBW)	7.4 ± 2.2	5.6 ± 2.0	< 0.001
PEEP (cm H_2_O)	11.8 ± 2.5	12.0 ± 3.5	0.653
Peak inspiratory pressure (cm H_2_O)	35.2 ± 5.4	30.5 ± 5.1	< 0.001
Mean airway pressure (cm H_2_O)	19.6 ± 3.8	17.0 ± 3.8	< 0.001
Dynamic compliance (ml/cm H_2_O)	19.9 ± 6.5	18.9 ± 8.5	0.520
Total respiratory rate (breaths/min)	20.3 ± 5.4	14.4 ± 3.5	< 0.001
Spontaneous respiratory rate (breaths/min)	4 (1–9)	0 (0–3)	< 0.001
Minute ventilation (L/min)	8.9 ± 2.5	4.5 ± 1.6	< 0.001
Arterial blood gas from day 1 to day 3 on ECMO			
pH	7.42 ± 0.08	7.44 ± 0.08	0.286
PaCO_2_ (mm Hg)	38.6 ± 6.5	38.1 ± 4.7	0.639
PaO_2_ (mm Hg)	102.2 ± 65.9	96.1 ± 39.5	0.489
Saturation (%)	94.8 ± 3.3	95.5 ± 2.9	0.240
PaO_2_/FiO_2_ (mm Hg)	151 (123–212)	189 (140–242)	0.921
SOFA score from day 1 to day 3 on ECMO	10.7 ± 2.2	9.2 ± 2.2	0.001
ECMO complications, *n* (%)	9 (22%)	34 (30.6%)	0.292
Duration of ECMO (days)	7.7 (4.7–11.5)	9.9 (5.9–15.8)	0.287
Duration of mechanical ventilator (days)	15.4 (11.8–34)	22.9 (12.4–39.8)	0.291
Length of ICU stay (days)	19 (10–43)	27 (16–43)	0.182
Length of hospital stay (days)	29 (13–63)	41 (24–65.5)	0.130
ECMO-free days on day 28	0 (0–18.2)	10.1 (0–19.3)	0.075
Ventilator-free days on day 28	0 (0–0)	0 (0–8.5)	0.311
Ventilator-free days on day 60	0 (0–20.4)	8.3 (0–40.5)	0.04
Hospital mortality, *n* (%)	29 (70.7%)	52 (46.8%)	0.004

Data are presented as mean ± standard deviation, count or median (interquartile range)

*ECMO* extracorporeal membrane oxygenation, *FiO*_*2*_ fraction of inspired oxygen, *ICU* intensive care unit, *MP* mechanical power, *PaCO*_*2*_ partial pressure of carbon dioxide in arterial blood, *PaO*_*2*_ partial pressure of oxygen in arterial blood, *PBW* predicted body weight, *PEEP* positive end-expiratory pressure, *SOFA* Sequential Organ Failure Assessment

**Table 3:** Ventilator settings, clinical variables, and outcomes as a function of mechanical power/compliance during ECMO

Variables	MP/Compliance during the first 3 days of ECMO		*p*
	High (*n* = 88) (> 0.53 J/min/ml/cm H_2_O)	Low (*n* = 64) (≤ 0.53 J/min/ml/cm H_2_O)	
Ventilator settings before ECMO			
MP (J/min)	23.8 ± 8.7	23.7 ± 10.1	0.990
MP/PBW (× 10^−3^ J/min/kg)	422 ± 158	407 ± 186	0.583
MP/Compliance (J/min/ml/cm H_2_O)	1.48 ± 0.85	0.98 ± 0.49	< 0.001
Tidal volume (ml/kg PBW)	7.5 ± 2.4	8.0 ± 2.3	0.193
PEEP (cm H_2_O)	12.2 ± 2.8	11.9 ± 2.7	0.539
Peak inspiratory pressure (cm H_2_O)	35.7 ± 7.0	31.7 ± 4.8	< 0.001
Mean airway pressure (cm H_2_O)	19.8 ± 4.3	17.3 ± 3.9	0.001
Dynamic compliance (ml/cm H_2_O)	19.3 ± 9.4	27.1 ± 12.2	< 0.001
Total respiratory rate (breaths/min)	25.2 ± 7.2	22.8 ± 6.5	0.042
Spontaneous respiratory rate (breaths/min)	2 (0–8)	0 (0–6)	0.197
Minute ventilation (L/min)	10.4 ± 3.6	10.8 ± 3.9	0.536
Arterial blood gas before ECMO			
pH	7.27 ± 0.15	7.28 ± 0.13	0.627
PaCO_2_ (mm Hg)	55.5 ± 21.6	48.1 ± 14.3	0.014
PaO_2_ (mm Hg)	74.4 ± 42.8	71.7 ± 35.0	0.687
Saturation (%)	83.7 ± 15.4	85.5 ± 15.5	0.493
PaO_2_/FiO_2_ (mm Hg)	60.7 (51.6–83)	67.4 (52.9–93.5)	0.851
SOFA score before ECMO	11.4 ± 3.2	9.9 ± 2.9	0.007
Ventilator settings from day 1 to day 3 on ECMO			
MP (J/min)	14.2 ± 6.8	8.7 ± 2.9	< 0.001
MP/PBW (× 10^−3^ J/min/kg)	249 ± 124	149 ± 51	< 0.001
MP/Compliance (J/min/ml/cm H_2_O)	0.99 ± 0.45	0.38 ± 0.10	< 0.001
Tidal volume (ml/kg PBW)	6.0 ± 2.3	6.0 ± 2.1	0.834
PEEP (cm H_2_O)	11.5 ± 3.2	12.9 ± 3.2	0.016
Peak inspiratory pressure (cm H_2_O)	34.8 ± 5.1	27.8 ± 3.1	< 0.001
Mean airway pressure (cm H_2_O)	18.4 ± 4.2	16.9 ± 3.8	0.027
Dynamic compliance (ml/cm H_2_O)	15.6 ± 6.7	24.1 ± 7.3	< 0.001
Total respiratory rate (breaths/min)	17.9 ± 4.1	13.1 ± 3.0	< 0.001
Spontaneous respiratory rate (breaths/min)	2 (0–5)	0 (0–3)	0.002
Minute ventilation (L/min)	6.3 ± 3.0	4.5 ± 1.7	< 0.001
Arterial blood gas from day 1 to day 3 on ECMO			
pH	7.43 ± 0.08	7.45 ± 0.08	0.192
PaCO_2_ (mm Hg)	39.2 ± 5.6	37.0 ± 4.7	0.016
PaO_2_ (mm Hg)	96.9 ± 54.0	99.6 ± 40.9	0.737
Saturation (%)	94.6 ± 3.4	96.3 ± 2.0	< 0.001
PaO_2_/FiO_2_ (mm Hg)	161.3 (125.5–208.8)	203 (152.3–250)	0.215
SOFA score from day 1 to day 3 on ECMO	10.1 ± 2.3	8.9 ± 2.2	0.002
ECMO complications, *n* (%)	26 (29.5%)	17 (26.6%)	0.688
Duration of ECMO (days)	10.6 (5.1–17.9)	7.9 (5.3–12.9)	0.054
Duration of mechanical ventilator (days)	24 (12–42.8)	20 (12–34.5)	0.622
Length of ICU stay (days)	26 (14–47)	23 (15.5–41)	0.806
Length of hospital stay (days)	44 (18.3–68.3)	38 (24.5–65.5)	0.577
ECMO-free days on day 28	0 (0–15.7)	17.3 (0–22.1)	< 0.001
Ventilator-free days on day 28	0 (0–0)	0 (0–15.1)	< 0.001
Ventilator-free days on day 60	0 (0–25.5)	30.7 (0–47.1)	< 0.001
Hospital mortality, *n* (%)	56 (63.6%)	19 (29.7%)	< 0.001

Data are presented as mean ± standard deviation, count or median (interquartile range)

*ECMO* extracorporeal membrane oxygenation, *FiO*_*2*_ fraction of inspired oxygen, *ICU* intensive care unit, *MP* mechanical power, *PaCO*_*2*_ partial pressure of carbon dioxide in arterial blood, *PaO*_*2*_ partial pressure of oxygen in arterial blood, *PBW* predicted body weight, *PEEP* positive end-expiratory pressure, *SOFA* Sequential Organ Failure Assessment

### Percentage changes in MP and its components after ECMO and correlation between MP and mortality

**Fig. 1:**
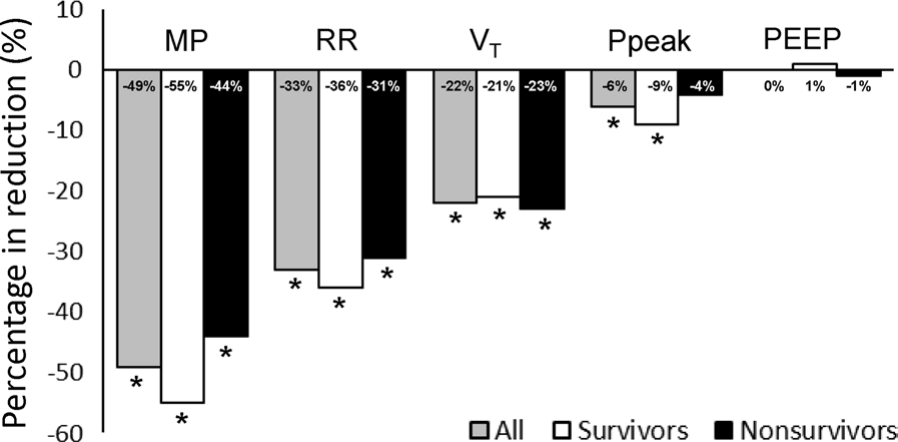
Difference in mean values of MP and its determinants before and during the first 3 days of ECMO. *p < 0.001 compared between the mean values before ECMO and during the first 3 days of ECMO. ECMO, extracorporeal membrane oxygenation; MP, mechanical power; PEEP, positive end-expiratory pressure; Ppeak, peak inspiratory pressure; RR, respiratory rate; *V*_T_, tidal volume

**Fig. 2:**
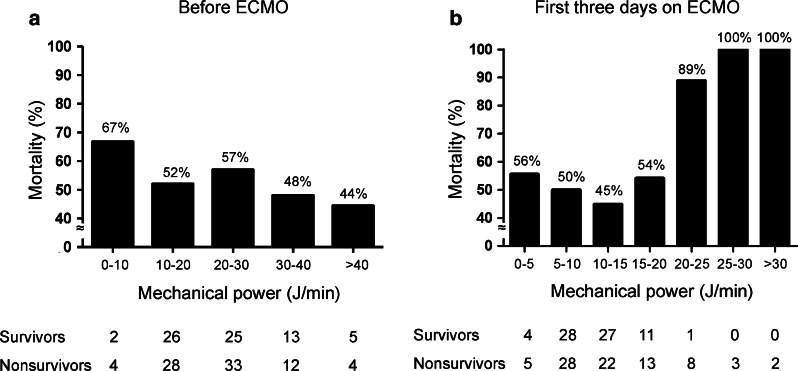
**a** Hospital mortality as a function of mean mechanical power before ECMO initiation. **b** During the first 3 days of ECMO

Following ECMO initiation, there was a significant reduction in MP among the overall population (49%, from 23.8 to 12.1 J/min, *p* < 0.001), survivors (55%, from 24.1 to 10.9 J/min, *p* < 0.001), and nonsurvivors (44%, from 23.5 to 13.1 J/min, *p* < 0.001). Following ECMO initiation, there was a pronounced decrease in total RR and *V*_T_ (33% and 22%, respectively, *p* < 0.001) with a less pronounced decrease in Ppeak (6%) and no change in PEEP in the overall population (Fig. 1). Hospital mortality was correlated with MP during the first 3 days of ECMO but not with the initial MP value before ECMO, and MP higher than 15.0 J/min during the first 3 days of ECMO showed consistently increasing trends in mortality. The hospital mortality was 89% among patients with MP exceeding 20 J/min during the first 3 days of ECMO and 49.3% among patients with MP of less than 20 J/min (Fig. 2a, b).

### Factors associated with hospital mortality

**Fig. 3:**
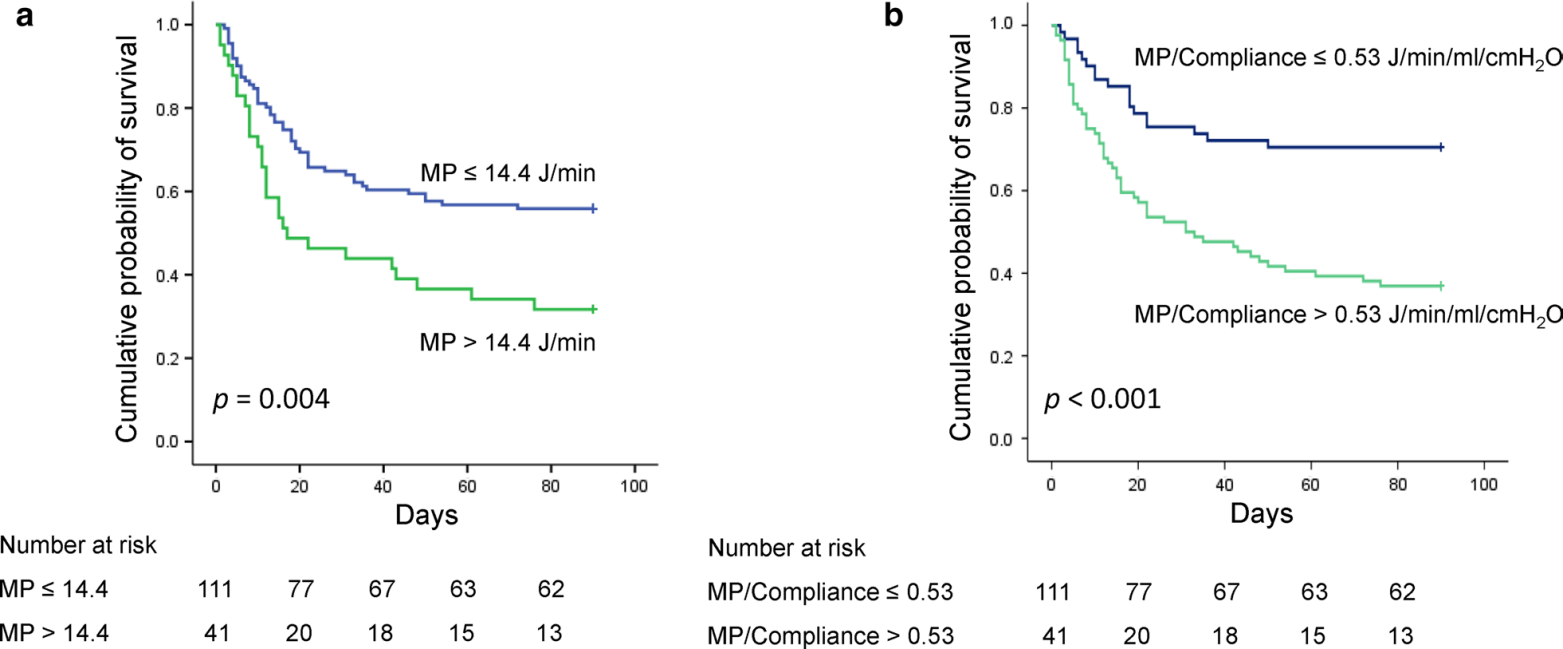
Kaplan–Meier 90-day survival curves of patients undergoing ECMO for severe acute respiratory distress syndrome, as stratified by the optimal cutoff value of **a** MP (14.4 J/min). **b** MP referenced to compliance (0.53 J/min/ml/cm H_2_O) during the first 3 days of ECMO. ECMO, extracorporeal membrane oxygenation; MP, mechanical power

After adjusting for significant confounding variables, Cox proportional hazard regression models revealed a number of factors that were significantly associated with 90-day hospital mortality: immunocompromised status, ARDS duration before ECMO, mean SOFA score from days 1–3 on ECMO, mean MP alone, mean MP referenced to PBW, and mean MP referenced to compliance from days 1–3 on ECMO. The risk of death was higher among patients with higher MP referenced to compliance during ECMO compared to those with higher MP alone or higher MP referenced to PBW (HR 2.289, 1.060, and 1.004, respectively, all *p* < 0.05) (Table 4). The overall 90-day survival rate was significantly higher among severe ARDS patients with mean MP ≦ 14.4 J/min from day 1 to 3 on ECMO than among those with mean MP > 14.4 J/min (53.2% vs. 29.3%, *p* = 0.004, log-rank test) (Fig. 3a), and the overall 90-day survival rate was significantly higher among severe ARDS patients with mean MP referenced to compliance ≦ 0.53 J/min/ml/cm H_2_O from day 1 to 3 on ECMO than among those with mean MP referenced to compliance > 0.53 J/min/ml/cm H_2_O (70.3% vs. 36.4%, *p* < 0.001, log-rank test) (Fig. 3b). Mean MP > 14.4 J/min during the first 3 days of ECMO was independently associated with higher hospital mortality (Adjusted HR 2.340 [95% CI 1.358–4.031]; *p* = 0.002) (Additional file 3: Table S3), and mean MP referenced to compliance > 0.53 J/min/ml/cm H_2_O during the first 3 days of ECMO was independently associated with higher hospital mortality (Adjusted HR 2.238 [95% CI 1.224–4.094]; *p* = 0.009) (Additional file 4: Table S4).

**Table 4:** Cox proportional hazard regression analysis of factors associated with 90-day hospital mortality

Variables	Univariate analysis		Multivariate analysis model 1		Multivariate analysis model 2		Multivariate analysis model 3	
	HR (95% CI)	*p*	HR (95% CI)	*p*	HR (95% CI)	*p*	HR (95% CI)	*p*
Age (with each year increase)	1.018 (1.004–1.033)	0.012						
Pulmonary cause	1.989 (1.211–3.216)	0.007						
Extrapulmonary cause	0.785 (0.475–1.296)	0.344						
Diabetes mellitus	0.622 (0.358–1.079)	0.091						
Chronic liver disease	2.085 (1.184–3.670)	0.011						
Immunocompromised status	2.242 (1.411–3.563)	0.001	2.564 (1.488–4.419)	0.001	2.674 (1.556–4.596)	< 0.001	2.554 (1.471–4.433)	0.001
ARDS duration before ECMO (with each hour increase)	1.002 (1.001–1.004)	< 0.001	1.002 (1.001–1.004)	0.003	1.002 (1.001–1.004)	0.003	1.001 (1.000–1.003)	0.074
SOFA score from day 1 to 3 on ECMO (with each point increase)	1.318 (1.178–1.476)	< 0.001	1.202 (1.067–1.355)	0.003	1.207 (1.074–1.356)	0.002	1.222 (1.084–1.377)	0.001
Tidal volume/PBW from day 1 to 3 on ECMO	1.001 (0.896–1.118)	0.992						
PEEP from day 1 to 3 on ECMO	0.945 (0.880–1.015)	0.120						
Peak inspiratory pressure from day 1 to 3 on ECMO	1.058 (1.019–1.100)	0.004						
Dynamic compliance from day 1 to 3 on ECMO	0.953 (0.924–0.984)	0.003						
Total respiratory rate from day 1 to 3 on ECMO	1.055 (1.003–1.109)	0.039						
MP from day 1 to 3 on ECMO	1.054 (1.017–1.093)	0.004	1.060 (1.018–1.104)	0.005				
MP/PBW from day 1 to 3 on ECMO (× 10^−3^ J/min/kg)	1.003 (1.001–1.005)	0.002			1.004 (1.002–1.007)	< 0.001		
MP/Compliance from day 1 to 3 on ECMO (J/min/ml/cm H_2_O)	3.142 (1.966–5.020)	< 0.001					2.289 (1.214–4.314)	0.010

*ARDS* acute respiratory distress syndrome, *CI* confidence interval, *ECMO* extracorporeal membrane oxygenation, *HR* hazard ratio, *MP* mechanical power, *PBW* predicted body weight, *PEEP* positive end-expiratory pressure, *SOFA* Sequential Organ Failure Assessment

Multivariate analysis models included age, pulmonary or extrapulmonary cause of ARDS, diabetes mellitus, chronic liver disease, immunocompromised status, ARDS duration before ECMO, mean SOFA score from day 1 to 3 on ECMO, and mean values of ventilatory parameters from day 1 to 3 on ECMO (tidal volume/PBW, PEEP, peak inspiratory pressure, dynamic compliance, total respiratory rate, MP, MP/PBW, and MP/Compliance)

Model 1: add mean MP from day 1 to 3 on ECMO

Model 2: add mean MP/PBW from day 1 to 3 on ECMO (× 10^−3^ J/min/kg)

Model 3: add mean MP/Compliance from day 1 to 3 on ECMO (J/min/ml/cm H_2_O)

## Discussion

The primary insight in this research was that MP alone, MP referenced to PBW, and MP referenced to compliance during the first 3 days of ECMO were all independently associated with hospital mortality. Among the ventilator settings variables, mechanical power referenced to compliance during the first 3 days of ECMO had the greatest predictive value for mortality.

ECMO facilitates the use of ultra-protective ventilation, which allows reductions in the contributors of energy load (i.e., MP) to mitigate further lung injury [1, 2]. Previous studies have reported that during the first 3 days of ECMO, higher PEEP [21] and lower driving pressure [16, 22] were independently associated with lower mortality. However, there was no clearly defined threshold indicating safe ventilator settings and MP values for patients with severe ARDS undergoing ECMO [2]. In the current study, we found that higher MP values during ECMO (but not before ECMO) were associated with increased mortality. In a Cox regression model, mean MP during the first 3 days of ECMO was independently associated with hospital mortality. Overall, our findings revealed that MP during ECMO could be considered a predictor of survival and should be taken into account in optimizing ventilation.

The energy load (MP) delivered to the lungs is not necessarily evenly distributed. The effects of MP on the respiratory system depend not only on the energy load itself but also on the pathophysiology of the lungs (e.g., functional lung size, proportion of inhomogeneity, and the recruitability) [4–6]. Therefore, MP should be adjusted for functional lung size to reflect the actual amount of energy expected to be delivered to the lungs. Specific power (SP), defined as power per ventilated lung unit or the power referenced to the dimension of the ventilated lung, should be considered for predicting VILI more precisely [9–11]. The concept of SP is important due to the fact that the “baby lung” of ARDS has smaller capacity functioning lung tissue for gas exchange, and the SP of the baby lung of ARDS far exceeds the lungs of a healthy adult when the same raw power was delivered [9, 23]. Concentrating the entire ventilation workload on a functioning baby lung that shrinks as it sustains injury increases its power exposure and the risk of entering the “VILI vortex”. Earlier intervention to minimize ventilatory demand and its associated MP to avoid progressing down the “VILI vortex” is necessary [11].

Respiratory system compliance is correlated directly with the amount of aerated lung available for tidal ventilation (functional lung size) in patients with ARDS, reflecting the dimension of baby lung [9, 11, 24]. Zhang et al*.* reported that MP referenced to compliance had highest discrimination in predicting mortality among all ventilator settings variables including MP alone in patients with ARDS [7]. Coppola et al. reported no causal relationship between MP alone and mortality, whereas both MP and transpulmonary MP referenced to respiratory system compliance or to the amount of well-aerated tissue were independently associated with ICU mortality of ARDS patients [8]. However, the above studies were predicated on baseline MP values referenced to compliance, they did not account for serial changes in MP referenced to compliance during the ICU stay and did not seek to determine whether the link between MP referenced to compliance and mortality was independent from other ventilator settings.

Patients with severe ARDS requiring ECMO tended to have more noninflated tissue (i.e., lower functional lung size), greater inhomogeneity, and greater lung recruitability [25]. There have been relatively few studies examining the effects of MP referenced to functional lung size on mortality in severe ARDS patients receiving ECMO. In the current study, we found that higher MP/Compliance values during ECMO were significantly associated with increased mortality. Cox regression models revealed that the risk of death estimates obtained using MP referenced to compliance were higher than those of MP alone or MP referenced to PBW, despite the fact that all three factors were independently associated with mortality (HR 2.289, 1.060, and 1.004, respectively, all *p* < 0.05). It indicated that functional lung size in ARDS patients is not always proportional to body weight [26], and is generally determined by the severity of the disease and is therefore better quantified by compliance [23, 24]. Our findings demonstrated that MP referenced to compliance is a superior representation of the actual amount of energy transmitted to the lungs and provided the most predictive value for hospital mortality among the ventilatory variables.

The most common cause of death among ARDS patients is multiorgan failure [27]. One international multicenter prospective study reported that extrapulmonary organ failure during ECMO had a significantly negative impact on 6-month mortality for patients with ARDS [19]. Our findings revealed that there was no significant difference between survivors and nonsurvivors in terms of MP and SOFA score before ECMO; however, MP and SOFA score were shown to decrease during the first 3 days of ECMO. SOFA score during the first 3 days of ECMO remained independently associated with hospital mortality. These findings indicated that ECMO could facilitate a further reduction in ventilator load (i.e., MP) in order to alleviate VILI by reducing the proinflammatory biotrauma response, thereby preventing multi-organ failure and improving survival [2, 28, 29]. Besides, an immunocompromised status was associated with lower survival, as reported in previous studies [19, 30]. The timing of ECMO initiation for severe ARDS has yet to be defined [1]; however, recent studies have also reported a link between ARDS duration before ECMO and mortality [19, 29].

This study was hindered by a number of limitations. First, this retrospective study was conducted in one tertiary care referral center with a high annual volume of patients requiring ECMO, thereby limiting generalizability. Second, ventilatory variables were recorded only once a day during the stay in the ICU and therefore do not necessarily represent dynamic changes in ventilator status, including fluctuations in MP during 24-h intervals. Third, we assessed functional lung size by means of PBW and compliance due to the retrospective study, but computed tomography scan of the lungs may be more accurate way to estimate amount of aerated remaining functional lung, lung inhomogeneity or the recruitability [7, 25]. However, computed tomography scan requires intra-hospital patient transfer from ICU to radiology department and the use of ECMO preclude widespread clinical use. Finally, our objective in this observational study was to identify the factors associated with mortality without considering issues pertaining to causality.

## Conclusions

Our findings revealed that MP referenced to compliance provided the most predictive value for hospital mortality among the ventilator settings variables. Defining safety limits to minimize VILI and decrease mortality in patients with severe ARDS undergoing ECMO may require larger randomized controlled trials to determine whether MP referenced to functional lung size, lung inhomogeneity, or recruitability is causally related to mortality.

## Supplementary Information

**Additional file 1: Table S1.** Ventilator settings before and during the first 3 days of ECMO between the earlier years and later years.

**Additional file 2: Table S2.** Ventilator settings parameters during the first 3 days of ECMO.

**Additional file 3: Table S3.** Cox proportional hazard regression analysis of factors associated with 90-day hospital mortality.

**Additional file 4: Table S4.** Cox proportional hazard regression analysis of factors associated with 90-day hospital mortality.

## Data Availability

The datasets used or analyzed in the study are available from the corresponding author on reasonable request.

## Abbreviations

ARDS: Acute respiratory distress syndrome
CI: Confidence interval
ECMO: Extracorporeal membrane oxygenation
HR: Hazard ratio
ICU: Intensive care unit
MP: Mechanical power
PBW: Predicted body weight
PEEP: Positive end-expiratory pressure
SOFA: Sequential organ failure assessment
SP: Specific power
*V*_T_: Tidal volume
Ppeak: Peak inspiratory pressure
∆*P*: Driving pressure
RR: Respiratory rate
VILI: Ventilator-induced lung injury
