# Supplementary material for: Mechanical power during extracorporeal membrane oxygenation and hospital mortality in patients with acute respiratory distress syndrome
Source: Crit Care. 2021 Jan 6;25:13. doi: 10.1186/s13054-020-03428-x (PMC7787230; doi:10.1186/s13054-020-03428-x)
Supplement: Supplementary file 1 — Additional file 1: Table S1. Ventilator settings before and during the first 3 days of ECMO between the earlier years and later years. [file 13054_2020_3428_MOESM1_ESM.docx]

**Table S1** Ventilator settings before and during the first 3 days of ECMO between the earlier years and later years

| Variables | 2006-2011 | 2012-2015 | *p* |
| --- | --- | --- | --- |
|  | (n = 77) | (n = 75) |  |
| PaO_2_/FiO_2_ (mm Hg) before ECMO | 56 (47-68) | 74 (57-107) | 0.001 |
| Ventilator settings before ECMO |  |  |  |
| MP (J/min) | 25.4 ± 9.1 | 22.1 ± 9.8 | 0.032 |
| MP/PBW (× 10^-3^J/min/kg) | 435 ± 152 | 398 ± 189 | 0.192 |
| MP/Compliance (J/min/ml/cm H_2_O) | 1.47 ± 0.89 | 1.07 ± 0.55 | 0.002 |
| Tidal volume (ml/kg PBW) | 7.6 ± 2.4 | 7.9 ± 2.4 | 0.535 |
| PEEP (cmH_2_O) | 12.6 ± 2.7 | 11.3 ± 2.8 | 0.004 |
| Peak inspiratory pressure (cm H_2_O) | 35.7 ± 7.3 | 32.1 ± 4.9 | <0.001 |
| Mean airway pressure (cm H_2_O) | 20.2 ± 4.3 | 17.0 ± 3.9 | <0.001 |
| Dynamic compliance (ml/cm H_2_O) | 21.1 ± 9.8 | 24.1 ± 12.5 | 0.111 |
| Total respiratory rate (breaths/min) | 25.1 ± 6.6 | 22.8 ± 7.1 | 0.041 |
| Spontaneous respiratory rate(breaths/min) | 2 (0-8) | 0 (0-6) | 0.372 |
| Minute ventilation (L/min) | 10.8 ± 3.6 | 10.4 ± 4.0 | 0.484 |
| PaO_2_/FiO_2_ (mm Hg) from day 1 to day 3 on ECMO | 165 (126-234) | 196 (141-240) | 0.094 |
| Ventilator settings from day 1 to day 3 on ECMO |  |  |  |
| MP (J/min) | 13.7 ± 6.0 | 10.5 ± 6.1 | 0.001 |
| MP/PBW (× 10^-3^J/min/kg) | 230 ± 101 | 183 ± 117 | 0.010 |
| MP/Compliance (J/min/ml/cm H_2_O) | 0.86 ± 0.50 | 0.60 ± 0.37 | <0.001 |
| Tidal volume (ml/kg PBW) | 6.5 ± 2.1 | 5.5 ± 2.2 | 0.005 |
| PEEP (cmH_2_O) | 10.6 ± 2.6 | 13.4 ± 3.3 | <0.001 |
| Peak inspiratory pressure (cm H_2_O) | 32.9 ± 6.0 | 30.5 ± 5.0 | 0.007 |
| Mean airway pressure (cm H_2_O) | 16.9 ± 3.4 | 18.5 ± 4.4 | 0.010 |
| Dynamic compliance (ml/cm H_2_O) | 18.3 ± 7.4 | 20.1 ± 8.7 | 0.168 |
| Total respiratory rate (breaths/min) | 16.7 ± 4.0 | 15.3 ± 4.8 | 0.047 |
| Spontaneous respiratory rate (breaths/min) | 2 (0-5) | 1 (0-4) | 0.134 |
| Minute ventilation (L/min) | 6.4 ± 2.6 | 4.9 ± 2.7 | 0.001 |

Data are presented as mean ± standard deviation or median (interquartile range)

*ECMO* extracorporeal membrane oxygenation, *FiO_2_* fraction of inspired oxygen, *MP* mechanical power, *PaO_2_* partial pressure of oxygen in arterial blood, *PBW* predicted body weight, *PEEP* positive end-expiratory pressure
