# Supplementary material for: Mechanical power during extracorporeal membrane oxygenation and hospital mortality in patients with acute respiratory distress syndrome
Source: Crit Care. 2021 Jan 6;25:13. doi: 10.1186/s13054-020-03428-x (PMC7787230; doi:10.1186/s13054-020-03428-x)
Supplement: Supplementary file 2 — Additional file 2: Table S2. Ventilator settings parameters during the first 3 days of ECMO. [file 13054_2020_3428_MOESM2_ESM.docx]

**Table S2** Ventilator settings parameters during the first 3 days of ECMO

| Characteristics | Day 1 | | | Day 2 | | | | Day 3 | | | |
| --- | --- | --- | --- | --- | --- | --- | --- | --- | --- | --- | --- |
|  | All | Survivors | Nonsurvivors | | All | Survivors | Nonsurvivors | | All | Survivors | Nonsurvivors |
| MP (J/min) | 12.5 ± 6.7 | 11.4 ± 5.3 | 13.5 ± 7.6 | | 11.6 ± 6.1 | 10.7 ± 4.5 | 12.5 ± 7.3 | | 11.5 ± 6.6 | 10.5 ± 5.2 | 12.3 ± 7.7 |
| MP/PBW (× 10^-3^J/min/kg) | 214 ± 121 | 194 ± 86 | 232 ± 143 | | 197 ± 106 | 182 ± 73 | 212 ± 129 | | 192 ± 107 | 176 ± 77 | 207 ± 128 |
| MP/Compliance (J/min/ml/cm H_2_O) | 0.76 ± 0.54 | 0.65 ± 0.4 | 0.85 ± 0.63 | | 0.71 ± 0.46 | 0.58 ± 0.32 | 0.83 ± 0.54 | | 0.70 ± 0.49 | 0.56 ± 0.35 | 0.84 ± 0.56 |
| Tidal volume (ml/kg PBW) | 6.1 ± 2.2 | 6.0 ± 2.1 | 6.2 ± 2.4 | | 5.9 ± 2.2 | 6.0 ± 2.0 | 5.8 ± 2.4 | | 5.9 ± 2.3 | 6.0 ± 2.1 | 5.8 ± 2.4 |
| PEEP (cm H_2_O) | 11.9 ± 3.3 | 12.3 ± 3.3 | 11.6 ± 3.3 | | 12.0 ± 3.4 | 12.3 ± 3.4 | 11.8 ± 3.5 | | 12.1 ± 3.5 | 12.5 ± 3.4 | 11.7 ± 3.5 |
| Peak inspiratory pressure (cm H_2_O) | 31.7 ± 5.7 | 30.9 ± 5.3 | 32.3 ± 6.0 | | 31.8 ± 5.9 | 30.6 ± 5.2 | 32.8 ± 6.4 | | 31.8 ± 6.5 | 30.4 ± 6.0 | 32.9 ± 6.8 |
| Mean airway pressure (cm H_2_O) | 17.7 ± 4.2 | 17.6 ± 4.1 | 17.7 ± 4.3 | | 17.7 ± 4.3 | 17.4 ± 4.0 | 18.0 ± 4.6 | | 17.6 ± 4.4 | 17.3 ± 3.8 | 17.9 ± 4.9 |
| Dynamic compliance (ml/cm H_2_O) | 19.2 ± 8.4 | 20.4 ± 8.3 | 18.2 ± 8.4 | | 18.9 ± 8.4 | 20.6 ± 7.4 | 17.3 ± 9.0 | | 19.2 ± 9.4 | 22.0 ± 9.5 | 16.5 ± 8.4 |
| Total respiratory rate (breaths/min) | 16.8 ± 6.1 | 16.1 ± 6.0 | 17.4 ± 6.2 | | 16.1 ± 5.5 | 15.2 ± 5.0 | 16.8 ± 5.9 | | 15.7 ± 5.5 | 14.4 ± 4.2 | 16.9 ± 6.2 |
| Spontaneous respiratory rate (breaths/min) | 0 (0-4) | 0 (0-4) | 0 (0-6) | | 0 (0-4) | 0 (0-2) | 0 (0-6) | | 0 (0-3) | 0 (0-1) | 0 (0-4) |
| Minute ventilation (L/min) | 5.9 ± 3.0 | 5.4 ± 2.5 | 6.3 ± 3.4 | | 5.5 ± 2.8 | 5.2 ± 2.1 | 5.7 ± 3.2 | | 5.3 ± 2.8 | 5.0 ± 2.3 | 5.6 ± 3.2 |

Data are presented as mean ± standard deviation or median (interquartile range)

*MP* mechanical power, *PBW* predicted body weight, *PEEP* positive end-expiratory pressure
