# Supplementary material for: Mechanical power during extracorporeal membrane oxygenation and hospital mortality in patients with acute respiratory distress syndrome
Source: Crit Care. 2021 Jan 6;25:13. doi: 10.1186/s13054-020-03428-x (PMC7787230; doi:10.1186/s13054-020-03428-x)
Supplement: Supplementary file 3 — Additional file 3: Table S3. Cox proportional hazard regression analysis of factors associated with 90-day hospital mortality. [file 13054_2020_3428_MOESM3_ESM.docx]

**Table S3** Cox proportional hazard regression analysis of factors associated with 90-day hospital mortality

| Factors | Hazard Ratio (95% CI) | *p* |
| --- | --- | --- |
| Univariate analysis |  |  |
| Age | 1.018 (1.004-1.033) | 0.012 |
| Pulmonary cause | 1.989 (1.211-3.216) | 0.007 |
| Extrapulmonary cause | 0.785 (0.475-1.296) | 0.344 |
| Diabetes mellitus | 0.622 (0.358-1.079) | 0.091 |
| Chronic liver disease | 2.085 (1.184-3.670) | 0.011 |
| Immunocompromised status | 2.242 (1.411-3.563) | 0.001 |
| ARDS duration before ECMO | 1.002 (1.001-1.004) | <0.001 |
| SOFA score from day 1 to 3 on ECMO | 1.318 (1.178-1.476) | <0.001 |
| Tidal volume/PBW from day 1 to 3 on ECMO | 1.001 (0.896-1.118) | 0.992 |
| PEEP from day 1 to 3 on ECMO | 0.945 (0.880-1.015) | 0.120 |
| Peak inspiratory pressure from day 1 to 3 on ECMO | 1.058 (1.019-1.100) | 0.004 |
| Dynamic compliance from day 1 to 3 on ECMO | 0.953 (0.924-0.984) | 0.003 |
| Total respiratory rate from day 1 to 3 on ECMO | 1.055 (1.003-1.109) | 0.039 |
| MP > 14.4 J/min from day 1 to 3 on ECMO | 1.943 (1.220-3.095) | 0.005 |
| Multivariate analysis |  |  |
| Immunocompromised status | 2.317 (1.369-3.923) | 0.002 |
| ARDS duration before ECMO | 1.002 (1.001-1.004) | 0.001 |
| SOFA score from day 1 to 3 on ECMO | 1.225 (1.092-1.374) | 0.001 |
| MP > 14.4 J/min from day 1 to 3 on ECMO | 2.340 (1.358-4.031) | 0.002 |

*ARDS* acute respiratory distress syndrome, *CI* confidence interval, *ECMO* extracorporeal membrane oxygenation, *MP* mechanical power, *PEEP* positive end-expiratory pressure, *PBW* predicted body weight, *SOFA* Sequential Organ Failure Assessment

Multivariate analysis model included age, pulmonary or extrapulmonary cause of ARDS, diabetes mellitus, chronic liver disease, immunocompromised status, ARDS duration before ECMO, mean SOFA score from day 1 to 3 on ECMO, and mean values of ventilatory parameters from day 1 to 3 on ECMO (tidal volume/PBW, PEEP, peak inspiratory pressure, dynamic compliance, total respiratory rate, MP)
